# Supplementary material for: A robotic sensory system with high spatiotemporal resolution for texture recognition
Source: Nat Commun. 2023 Nov 14;14:7121. doi: 10.1038/s41467-023-42722-4 (PMC10645869; doi:10.1038/s41467-023-42722-4)
Supplement: Supplementary file 1 — Supplementary Information [file 41467_2023_42722_MOESM1_ESM.pdf]

Supplementary Information for

A robotic sensory system with high spatiotemporal resolution for texture recognition

Ningning Bai[1,2,5], Yiheng Xue[3,5], Shuiqing Chen[3], Lin Shi[1], Junli Shi[1], Yuan Zhang[1], Xingyu Hou[1], Yu Cheng[1], Kaixi Huang[1], Weidong Wang[2], Jin Zhang[3], Yuan Liu[4], Chuan Fei Guo[1,*]

[1]Department of Materials Science and Engineering, Southern University of Science and Technology, Shenzhen 518055, China

[2]School of Mechano-Electronic Engineering, Xidian University, Xi'an 710071, China

[3]Department of Computer Science and Engineering, Southern University of Science and Technology, Shenzhen 518055, China

[4]Department of Physics and TcSUH, University of Houston, Houston TX 77204, USA

[5]These authors contributed equally: Ningning Bai, Yiheng Xue

*To whom correspondence should be addressed. E-mail: guocf@sustech.edu.cn

The file includes:

Supplementary Table 1. Information of the 20 textiles used in this work.

Supplementary Table 2. Top ten important extracted signal features.

Supplementary Table 3. Comparison of our sensing system based on the slip-sensor with reported sensory systems in terms of acquisition system and recognition accuracy.

Supplementary Fig. 1. SEM image showing the cross section of a human fingerprint replica.

Supplementary Fig. 2. Change in capacitance over 10,000 loading-release cycles under a peak pressure of 100 kPa, with a total test time of ~14.5 h.

Supplementary Fig. 3 Hysteresis curve of the slip-sensor during a loading-unloading cycle.

Supplementary Fig. 4. The capacitance of the slip-sensor at different pressures.

Supplementary Fig. 5. Stress-strain curves of PDMS, a soft PVA-H_3PO_4 film, and the PVA-H_3PO_4 film which is used as the ionic layer in our sensor.

Supplementary Fig. 6. Response to different microstructures of the sensor with fingerprint tip size of 25 μm.

Supplementary Fig. 7. Signals to the microstructures with a spacing of 15 μm at a sliding rate of 1.0 $mm \cdot s^{-1}$ under contact pressures of 2, 10, and 20 kPa.

Supplementary Fig. 8. Signal of the slip-sensor by sliding on fine textures with a spacing of 15 μm and a height of 6 μm at a sliding rate of 1 $mm \cdot s^{-1}$.

Supplementary Fig. 9. Recognition accuracy of surface structures with different feature spacings by five volunteer subjects.

Supplementary Fig. 10. Effect of elastic modulus on signal magnitudes.

Supplementary Fig. 11. SEM images of the 20 textiles under test.

Supplementary Fig. 12. Schematic design diagram of the digital circuit board.

Supplementary Fig. 13. Frequency-domain signals of the 20 textiles obtained using wavelet transform.

Supplementary Fig. 14. Recognition accuracy of the 20 textiles at a high sliding rate of 40 $mm \cdot s^{-1}$.

Supplementary Fig. 15. Time-domain signals of the slip-sensor corresponding to different textures under random sliding rates and pressures.

Supplementary Fig. 16. Change in acceleration of subject A when sliding on 20 textiles, with 200 times of sliding for each textile.

Supplementary Fig. 17. Change in acceleration of subject B when sliding on 20 textiles, with 100 times of sliding for each textile.

Supplementary Fig. 18. Change in acceleration of subject C when sliding on 20 textiles, with 100 times of sliding for each textile.

Supplementary Fig. 19. Recognition accuracies of the 20 textiles using a sensor without fingerprint.

Supplementary Fig. 20. The signal-to-noise ratio (SNR) and effective number of bits (ENOB) of the signal output of the slip-sensor.

Supplementary Fig. 21. Schematic for the preparation of the slip-sensor.

The supplementary note: recognition of microstructures by human subjects.

Supplementary Table 1. Information of the 20 textiles used in this work.

Number	Name and source	Type	Components
1	Blended double coat fabric	Blended fabric	40% Rayon, 46% Terylene, 14% Nylon
2	Pure wool fine grain coat	Pure wool	100% Wool
3	Khaki trench coat fabric	Pure cotton	100% Cotton
4	Blended linen fabric	Blended fabric	80% Linen, 20% Cotton
5	Polyester imitation cotton cloth	Blended fabric	70% Terylene, 30% Rayon
6	Twill cloth	Pure cotton	100% Cotton
7	Laser phantom color down jacket	Pure fabric	100% Terylene
8	Waterproof jacquard trench coat	Pure fabric	100% Nylon
9	Cross print plain fabric	Blended fabric	50% Cotton, 50% Terylene
10	Net yarn embroidery	Pure fabric	100% Polyester
11	Super lightweight nylon fabric	Pure fabric	100% Nylon
12	Seersucker	Pure fabric	100% Terylene
13	Striped blended cloth	Blended fabric	75% Cotton, 25% Spandex
14	Corduroy	Pure cotton	100% Cotton
15	Jacquard fabric	Blended fabric	65% Polyester, 33% Viscose, 2% Lycra
16	SUSTANS	Blended fabric	Novel bio-elastic short fiber
17	Super imitation cotton	Blended fabric	70% Polyester, 30% Cotton
18	Wool blended fabric	Blended fabric	55% Nylon, 25% Cotton, 20% Wool
19	Stretch blend cloth	Blended fabric	65% Spandex, 35% Polyester
20	Twill	Pure fabric	100% Polyester

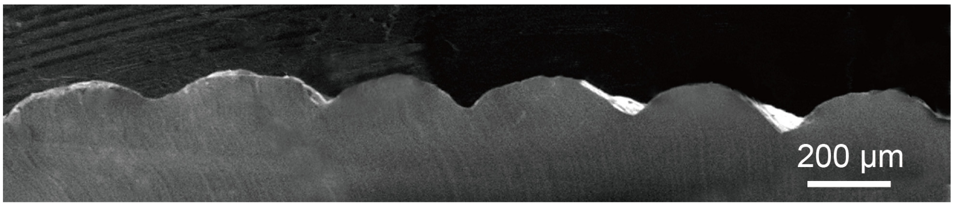

Supplementary Fig. 1. SEM image showing the cross section of a human fingerprint replica.

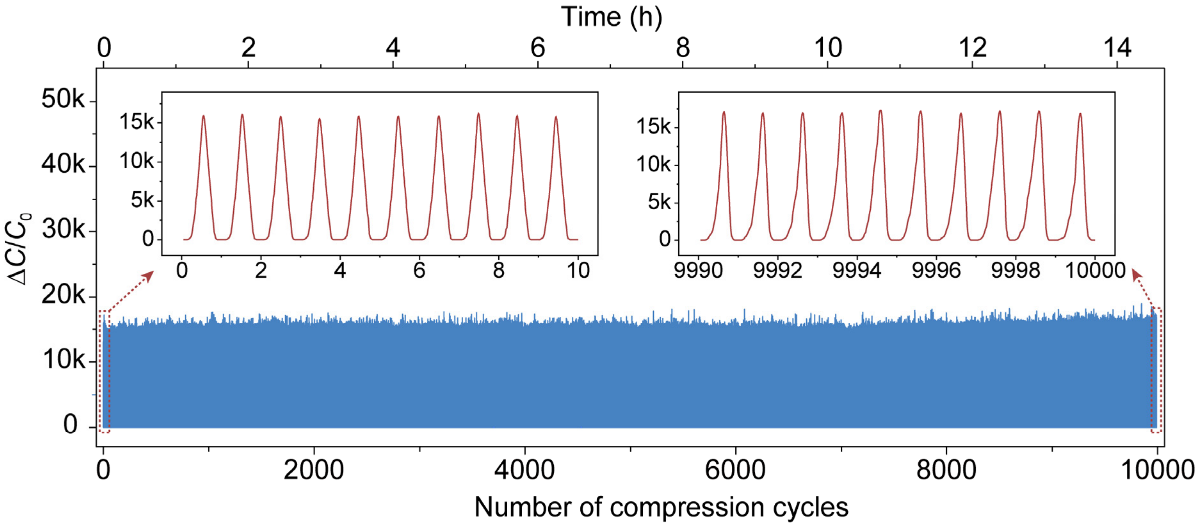

Supplementary Fig. 2. Change in capacitance over 10,000 loading-release cycles under a peak pressure of 100 kPa, with a total test time of ~14.5 h.

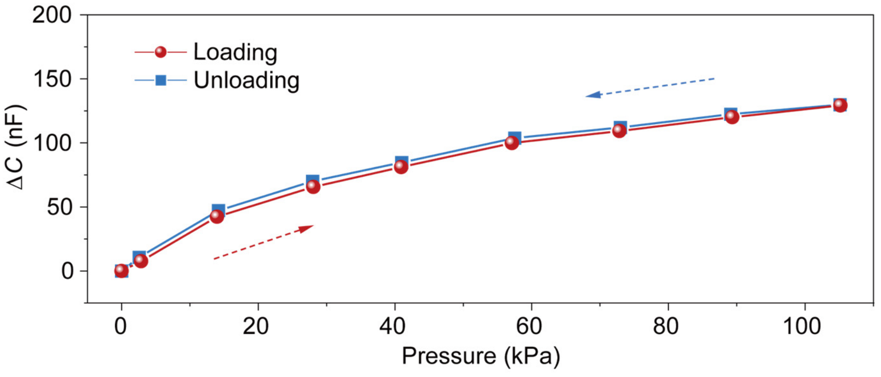

Supplementary Fig. 3 Hysteresis curve of the slip-sensor during a loading-unloading cycle.

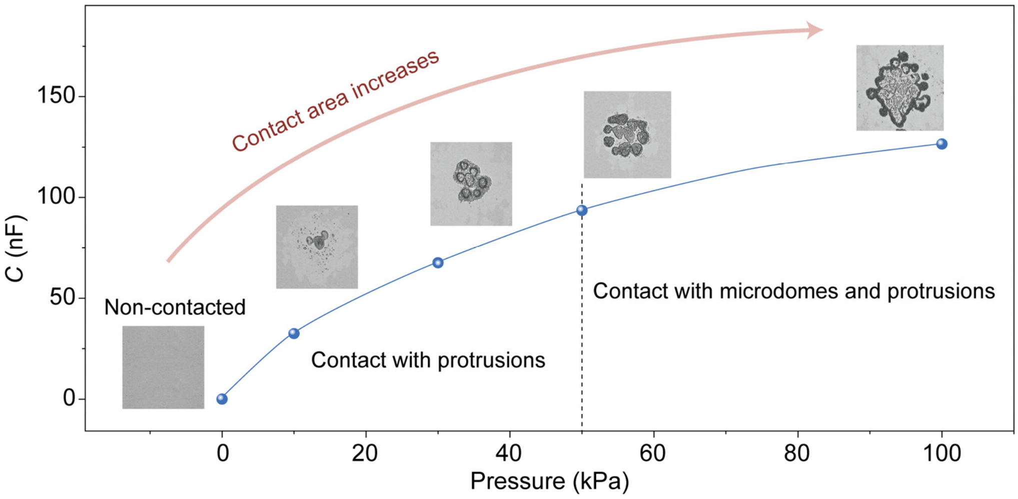

Supplementary Fig. 4. The Capacitance of the slip-sensor at different pressures. The inset pictures show the interfacial contact area under different pressures.

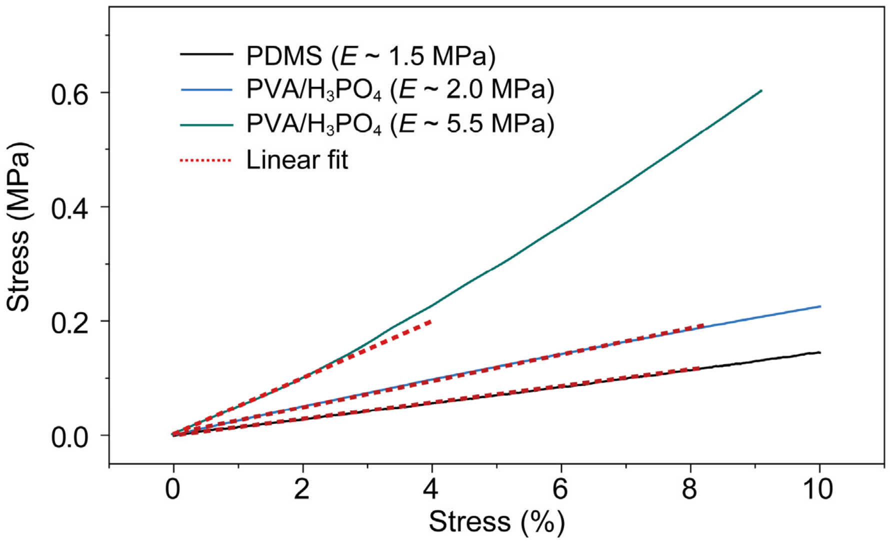

Supplementary Fig. 5. Stress-strain curves of PDMS, a soft PVA-H_3PO_4 film, and the PVA-H_3PO_4 film which is used as the ionic layer in our sensor. The specimens exhibit Young's moduli of 1.5, 2.0, and 5.5 MPa, respectively.

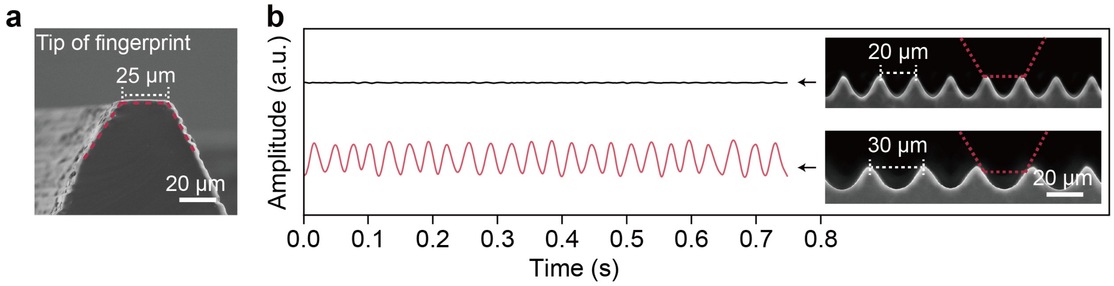

Supplementary Fig. 6. Response to different microstructures of the sensor with fingerprint tip size of 25 μm. a SEM image of an artificial fingerprint with a ridge tip width of 25 μm. **b** Signals of the slip-sensor with a fingerprint tip width of 25 μm when sliding on surface features with spacings of 20 and 30 μm at a sliding rate of 1 mm·s^{-1}. Insets are SEM images of the surface structures. Here, "a. u." signifies "arbitrary unit".

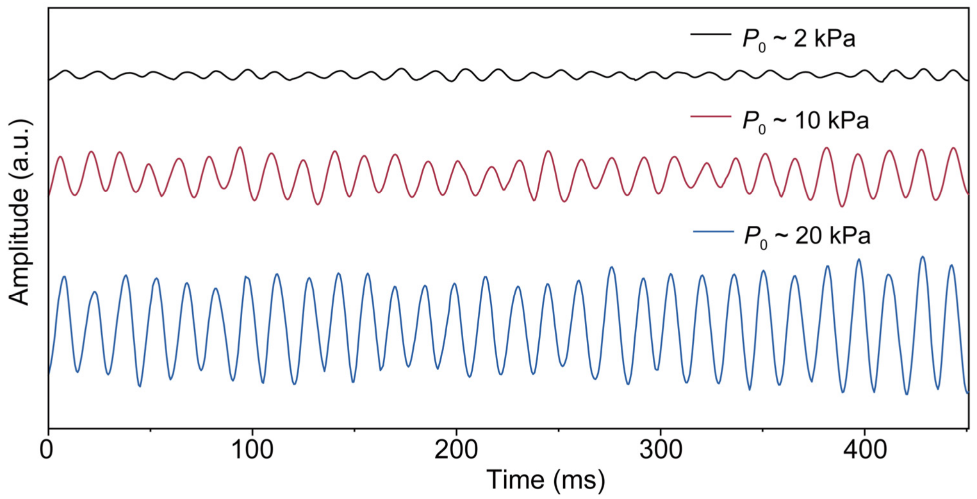

Supplementary Fig. 7. Signals to the microstructures with a spacing of 15 μm at a sliding rate of 1.0 mm·s^{-1} under contact pressures of 2, 10, and 20 kPa.

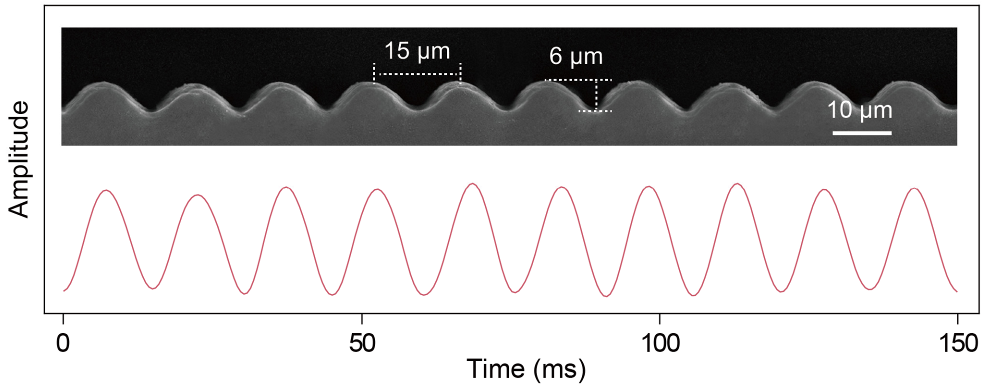

Supplementary Fig. 8. Signal of the slip-sensor by sliding on fine textures with a spacing of 15 μm and a height of 6 μm at a sliding rate of 1 mm·s^{-1}. The inset shows an SEM image of the texture.

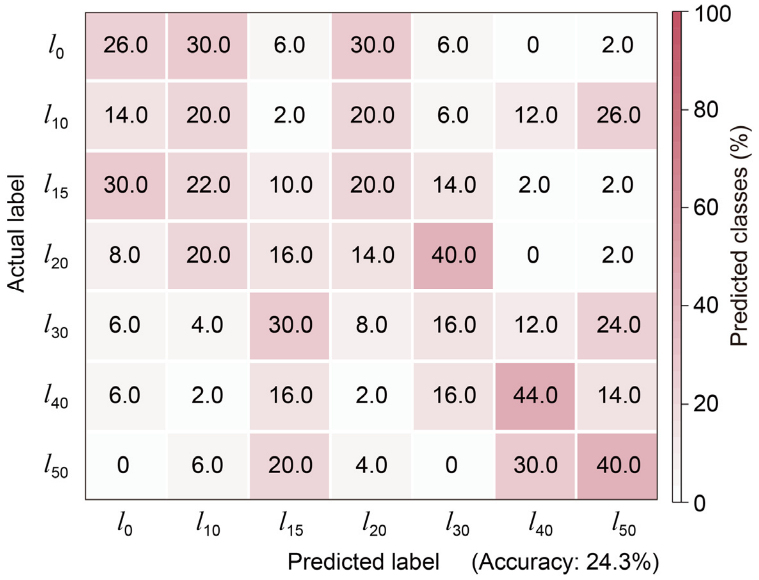

Supplementary Fig. 9. Recognition accuracy of surface structures with different feature spacings by five volunteer subjects. The labels l_0 represents nonstructured surface; and l_{10}, l_{15}, l_{20}, l_{30}, l_{40}, and l_{50} represent the textures with feature spacing of 10, 15, 20, 30, 40, and 50 μm, respectively.

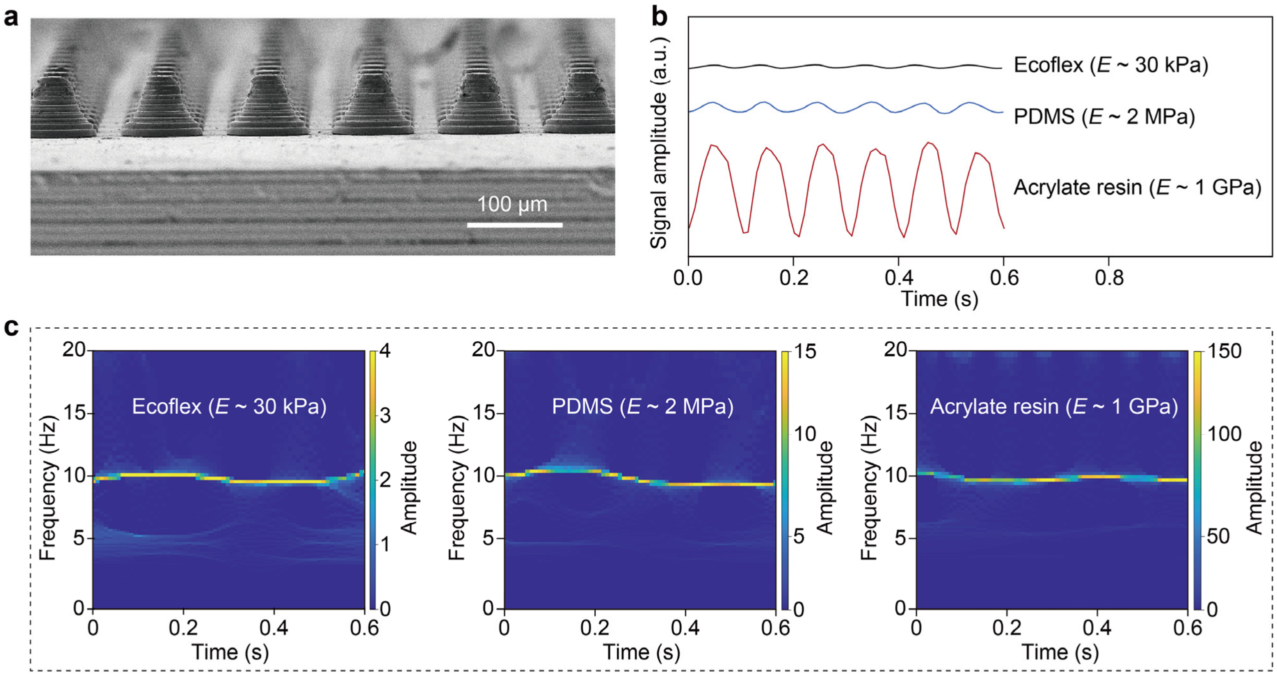

Supplementary Fig. 10. Effect of elastic modulus on signal magnitudes. **a** SEM image textures made of acrylate resin, and two other samples with the same structure made of Ecoflex and PDMS are also prepared. **b** Signals of the slip-sensor when the sensor slides over the surfaces of Ecoflex ($E \sim 30$ kPa), PDMS ($E \sim 2$ MPa), and resin ($E \sim 1$ GPa) structures. Sliding rate is 1 mm·s^{-1}. **c** Frequency-domain signals corresponding to panel (b).

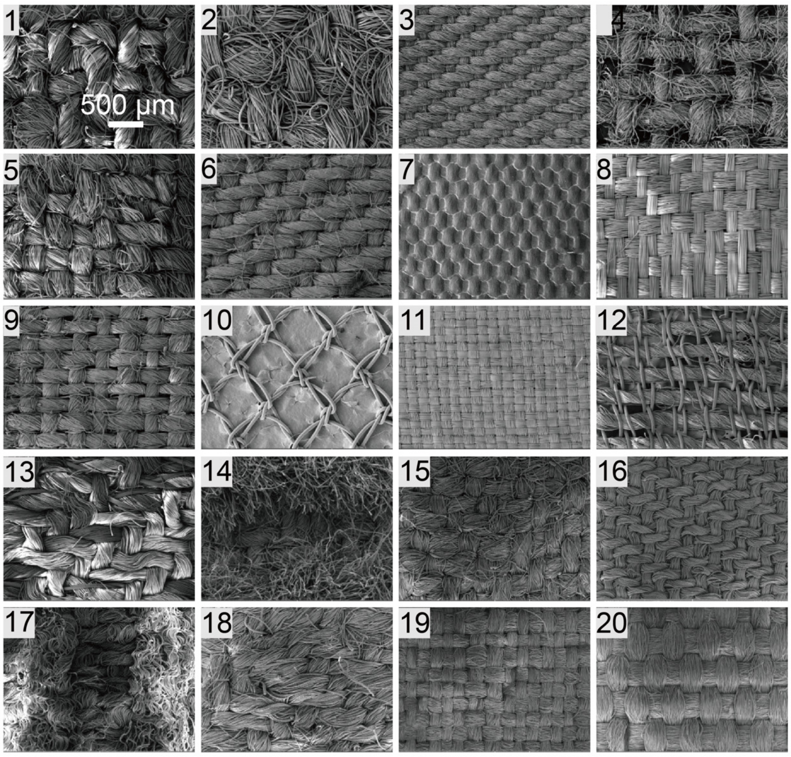

Supplementary Fig. 11. SEM images of the 20 textiles.

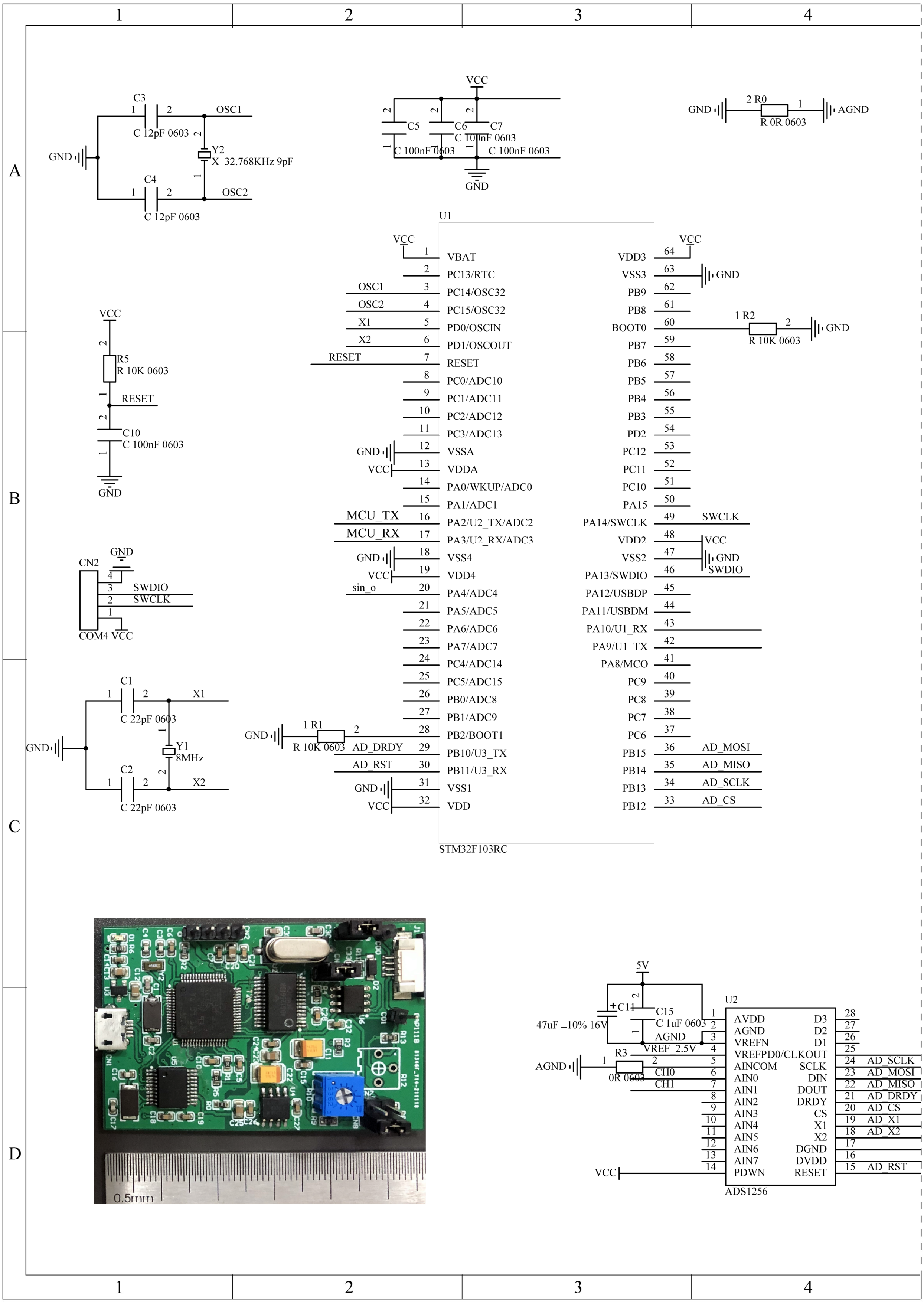
1
2
3
4
A
B
C
D
C3
C 12pF 0603
OSC1
GND
Y2
X_32.768KHz 9pF
C4
C 12pF 0603
OSC2
VCC
C5
C6
C7
C 100nF 0603
C 100nF 0603
C 100nF 0603
GND
2 R0
R 0R 0603
AGND
U1
VBAT
PC13/RTC
PC14/OSC32
PC15/OSC32
PD0/OSCIN
PD1/OSCOUT
RESET
PC0/ADC10
PC1/ADC11
PC2/ADC12
PC3/ADC13
VSSA
VDDA
PA0/WKUP/ADC0
PA1/ADC1
PA2/U2_TX/ADC2
PA3/U2_RX/ADC3
VSS4
VDD4
PA4/ADC4
PA5/ADC5
PA6/ADC6
PA7/ADC7
PC4/ADC14
PC5/ADC15
PB0/ADC8
PB1/ADC9
PB2/BOOT1
PB10/U3_TX
PB11/U3_RX
VSS1
VDD
VDD3
VSS3
PB9
PB8
BOOT0
PB7
PB6
PB5
PB4
PB3
PD2
PC12
PC11
PC10
PA15
PA14/SWCLK
VDD2
VSS2
PA13/SWDIO
PA12/USBDP
PA11/USBDM
PA10/U1_RX
PA9/U1_TX
PA8/MCO
PC9
PC8
PC7
PC6
PB15
PB14
PB13
PB12
STM32F103RC
X1
X2
MCU_TX
MCU_RX
sin_o
AD_DRDY
AD_RST
SWCLK
SWDIO
AD_MOSI
AD_MISO
AD_SCLK
AD_CS
1 R2
R 10K 0603
1 R1
R 10K 0603
R5
R 10K 0603
C10
C 100nF 0603
CN2
COM4
C1
C 22pF 0603
Y1
8MHz
C2
C 22pF 0603
5V
C14
47uF ±10% 16V
C15
C 1uF 0603
AGND
VREF_2.5V
R3
0R 0603
CH0
CH1
U2
AVDD
AGND
VREFN
VREFPD0/CLKOUT
AINCOM
AIN0
AIN1
AIN2
AIN3
AIN4
AIN5
AIN6
AIN7
PDWN
D3
D2
D1
SCLK
DIN
DOUT
DRDY
CS
X1
X2
DGND
DVDD
RESET
AD_X1
AD_X2
ADS1256
0.5mm

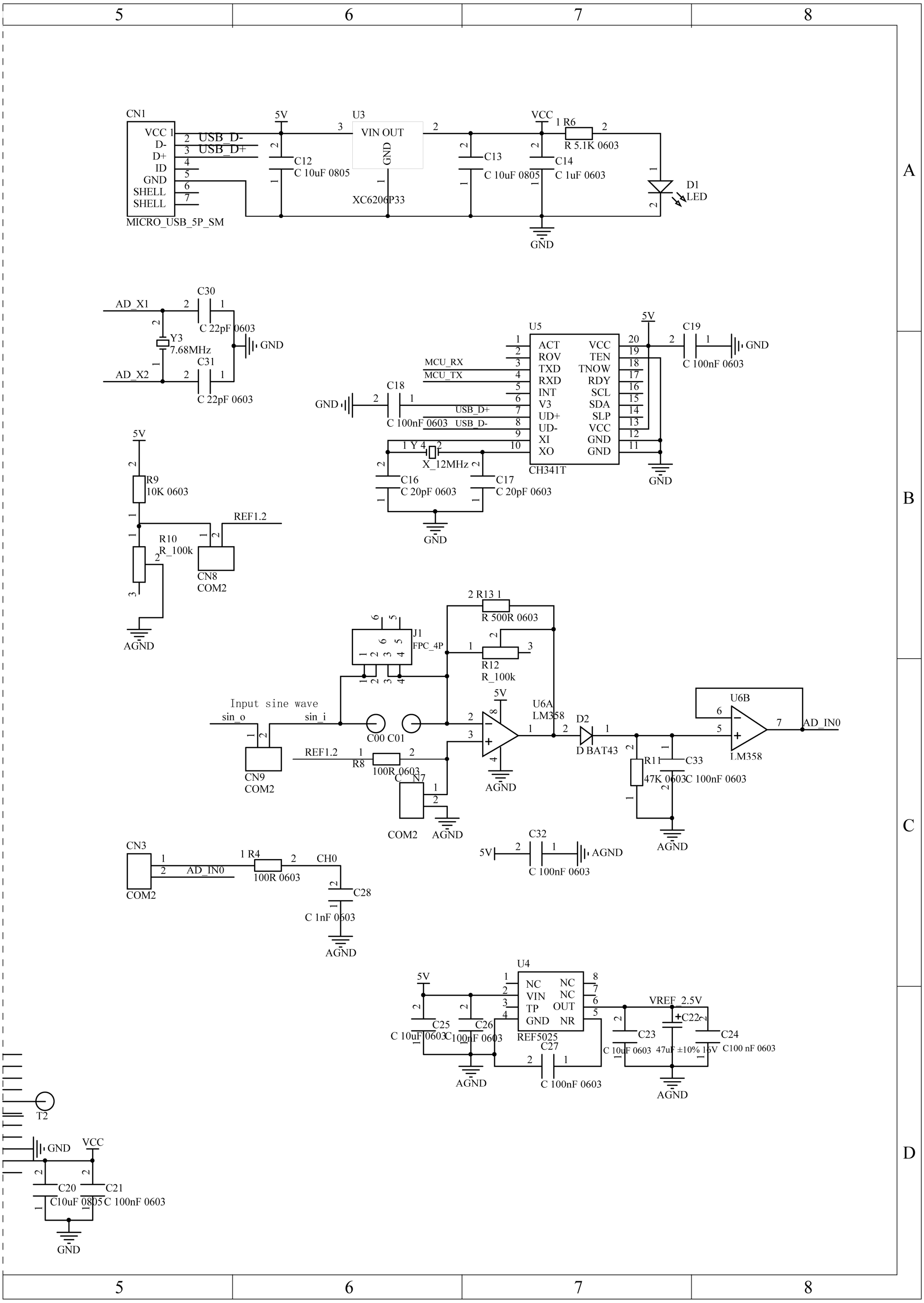

Supplementary Fig. 12. Schematic design diagram of the digital circuit board. The inset is a digital photograph of the circuit board.

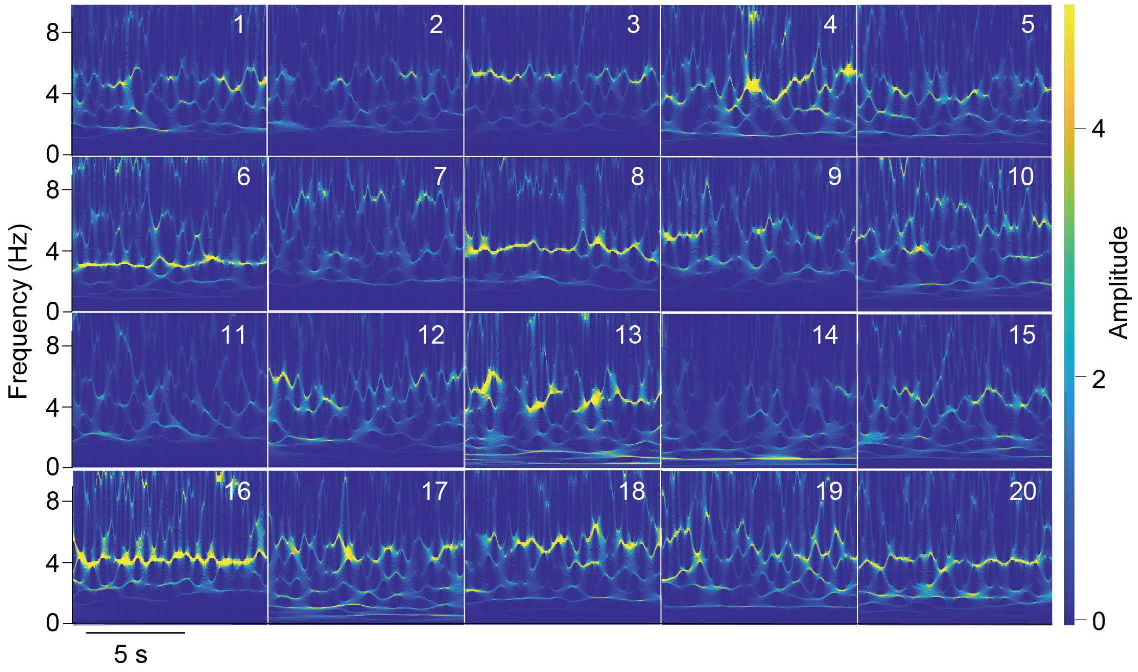

Supplementary Fig. 13. Frequency-domain signals of the 20 textiles obtained using wavelet transform. The sliding rate is 2 $mm{\cdot}s^{-1}$.

Supplementary Table 2. Top ten important extracted signal features

	Feature Name	Description
1	Range Count	Count of values
2	Sequence Length	Length of the feature sequence
3	Mean Quantile Change	Average change in quantiles
4	Mean Change	Mean change in feature values
5	Absolute Quantile Variance	Variance in quantile changes (absolute)
6	Sequence Complexity	Complexity of the sequence (not normalized)
7	0.6 Upper Limit Abs Quantile Variance	Variance in quantile changes (absolute, 0.6 upper limit)
8	0.6 Upper Limit Quantile Variance	Variance in quantile changes (0.6 upper limit)
9	Quantile Variance	Variance in quantile changes
10	Fourier Variance	Variance in Fourier-transformed values

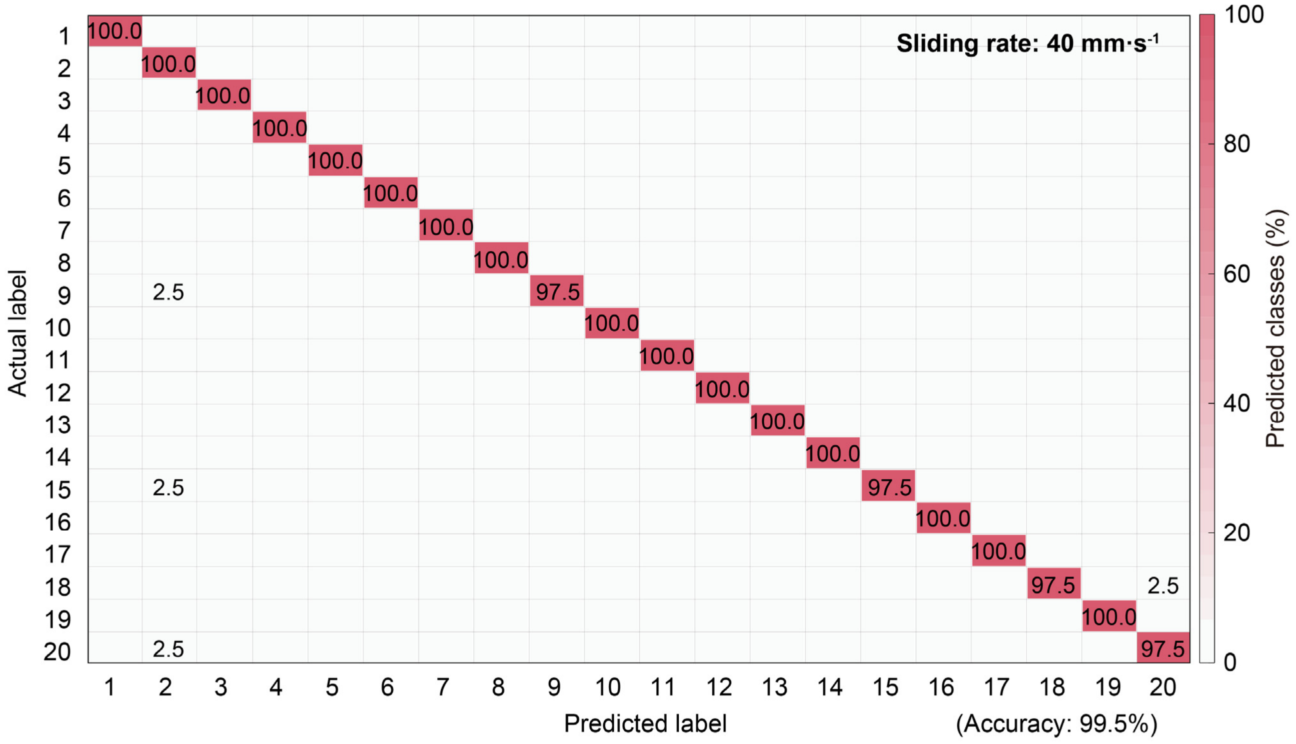

Supplementary Fig. 14. Recognition accuracy of the 20 textiles using the data collected at a high sliding rate of 40 mm·s^{-1}.

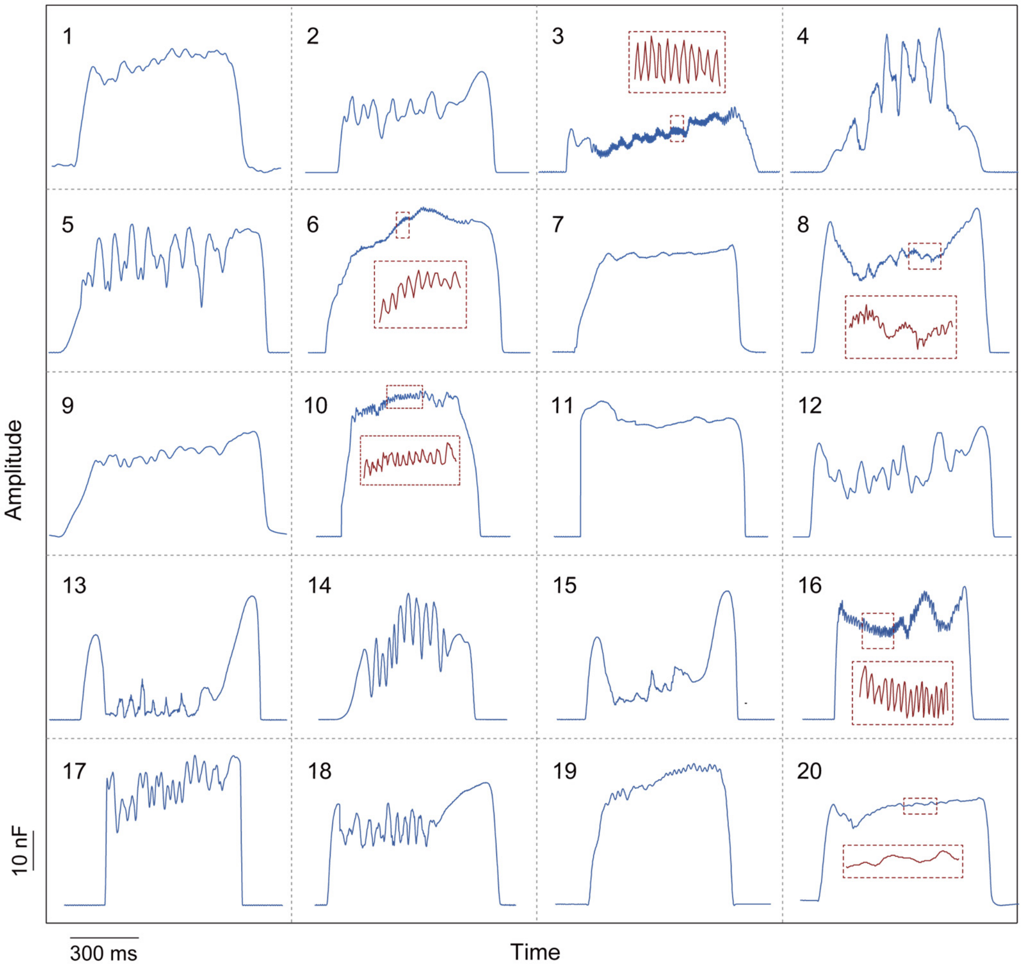

Supplementary Fig. 15. Time-domain signals of the slip-sensor corresponding to different textures under random sliding rates and pressures.

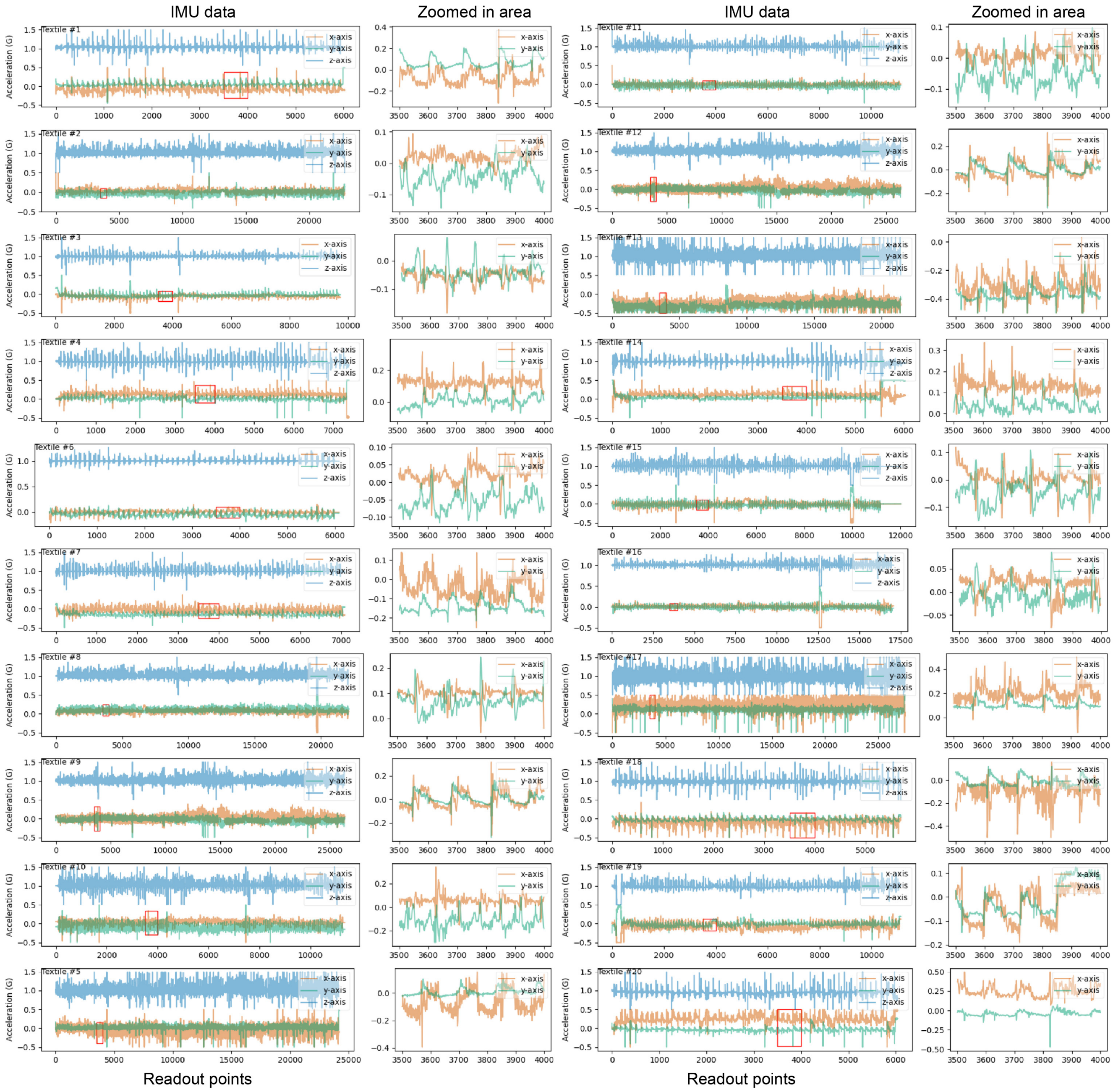

Supplementary Fig. 16. Change in acceleration of subject *A* when sliding on 20 textiles, with 200 times of sliding for each textile. The chaotic acceleration in the *x*-*y* plane corresponding to multiple sliding for each textile represents that the sliding velocities are variable and random.

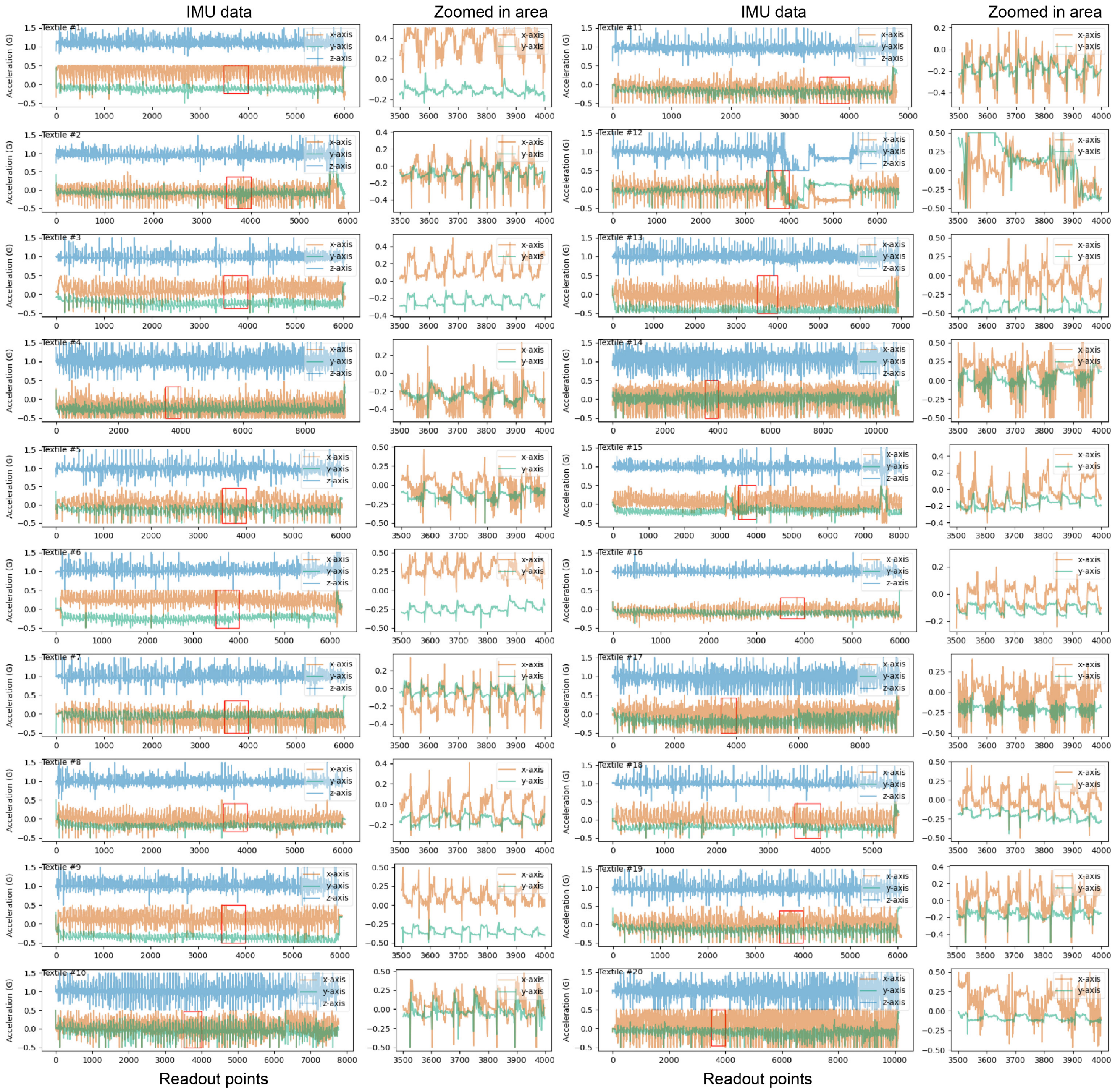

Supplementary Fig. 17. Change in acceleration of subject *B* when sliding on 20 textiles, with 100 times of sliding for each textile. The chaotic acceleration in the *x*-*y* plane corresponding to multiple sliding for each textile represents that the sliding velocities are variable and randon.

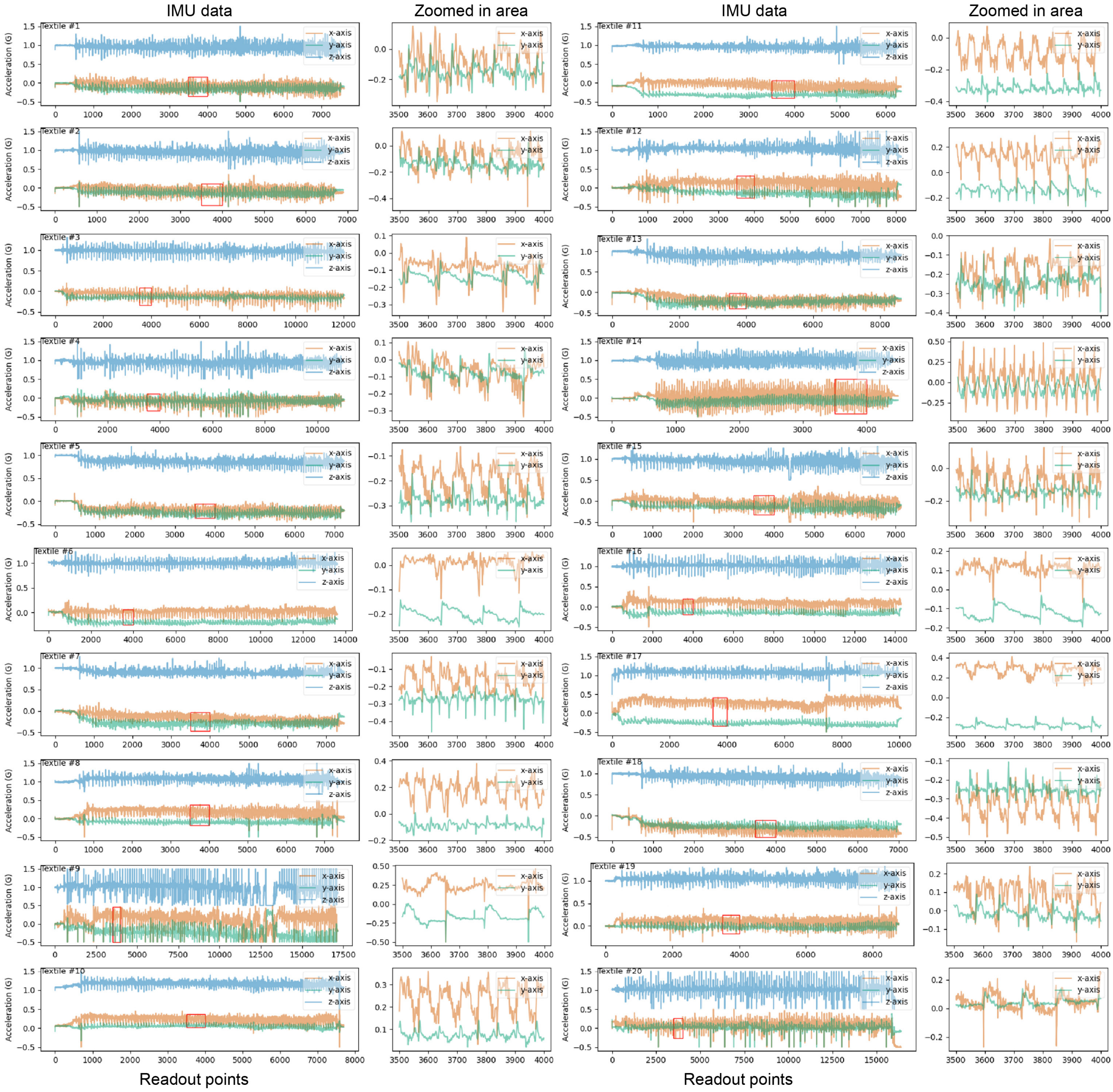

Supplementary Fig. 18. Change in acceleration of subject *C* when sliding on 20 textiles, with 100 times of sliding for each textile. The chaotic acceleration in the *x*-*y* plane corresponding to multiple sliding for each textile represents that the sliding velocities are variable and randon.

Supplementary Table 3. Comparison of our sensing system based on the slip-sensor with reported sensory systems in terms of acquisition system and recognition accuracy.

SA signal mimicking	FA signal mimicking	No. of acquisition systems	No. of recognized objects	Recognition accuracy	Reference
Piezoresistive sensor	Triboelectric sensor	Two sets	12	99.1%	*Nano Lett.* 2019, 19, 3305-3312
Piezoresistive sensor	Piezoelectric sensor	Two sets	20	99.1%	*Nat. Electron.* 2021, 4, 429-438
Triboelectric generator	Optical sensor	Two sets	16	94.1%	*Nano Lett.* 2022, 22, 7275-7283
Piezoresistive sensor	Triboelectric generator	Two sets	9	94.99%	*Nano Energy* 2022, 96, 107137
Piezoresistive sensor	Piezoelectric sensor	Two sets	8	98.95%	*npj Flex. Electron.* 2022, 6, 45
Piezoresistive sensor	Triboelectric generator	Two sets	20	98.9%	*Matter* 2022, 5, 1481-1501
**A single iontronic slip-sensor**		One set	20	98.6%	**Our work**

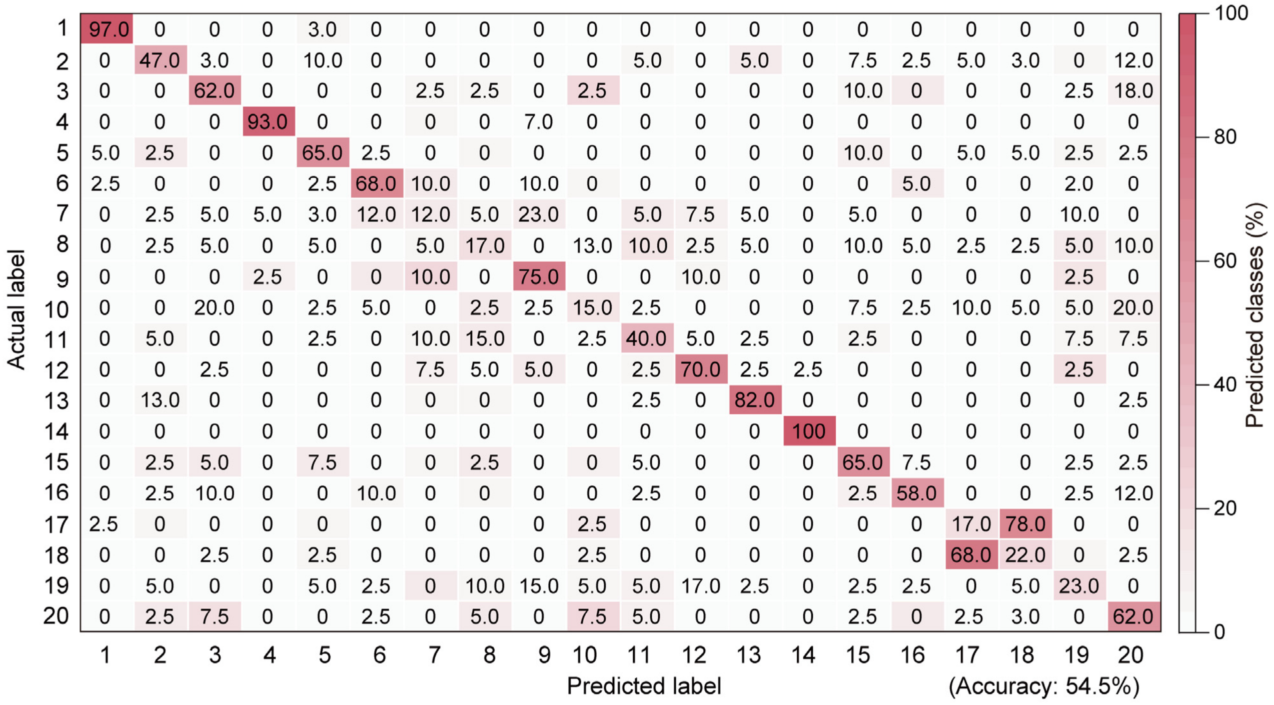

Supplementary Fig. 19. Recognition accuracies of the 20 textiles using the sensor without fingerprint at a sliding rate of 2 mm·s^{-1}.

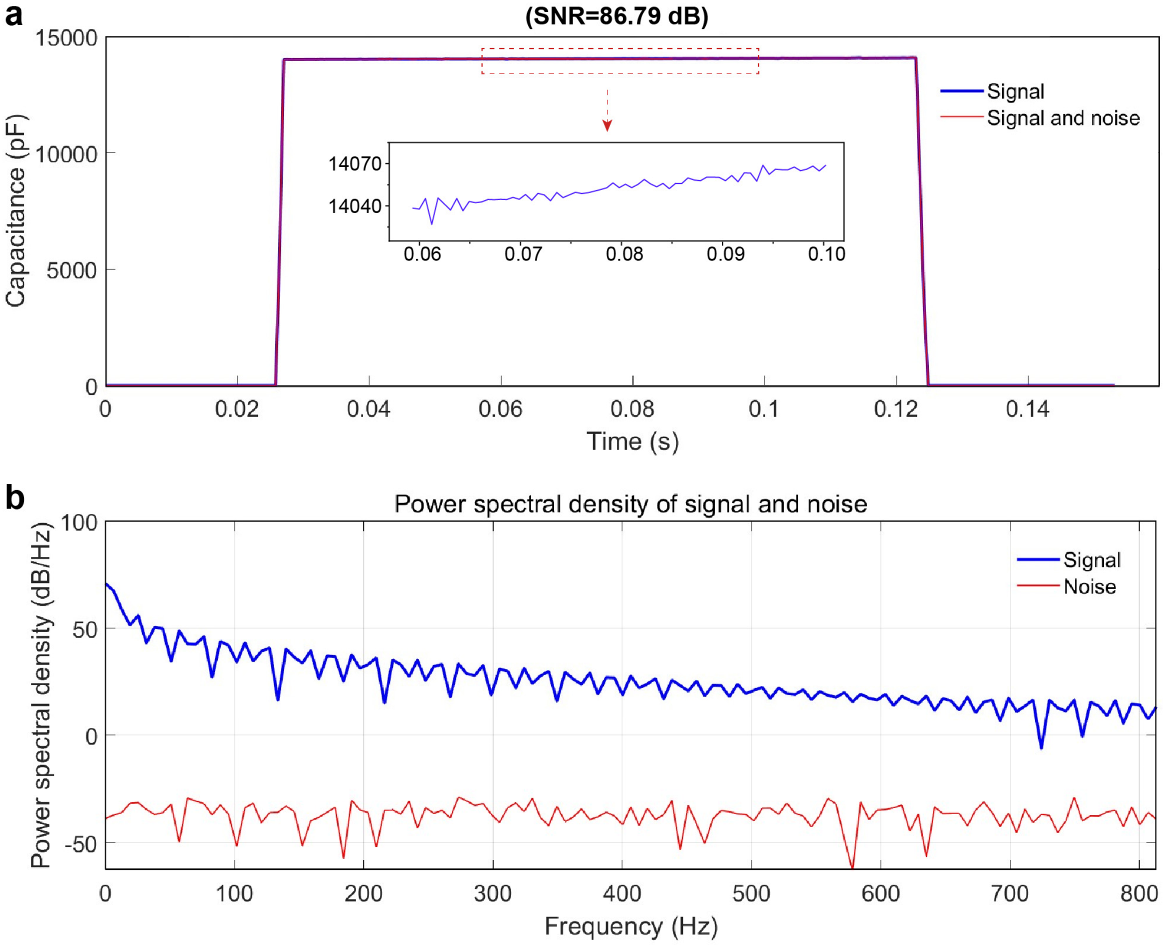

Supplementary Fig. 20. The signal-to-noise ratio (SNR) and effective number of bits (ENOB) of the signal output of the slip-sensor. (a) Signal and noise of the slip-sensor under a pressure of 50 kPa at a high sampling frequency of 1626 Hz; (b) Power spectral density of the signal and noise. The ENOB value is determined to be 14.12 bits by the equation: ENOB = (SNR–1.76)/6.02.

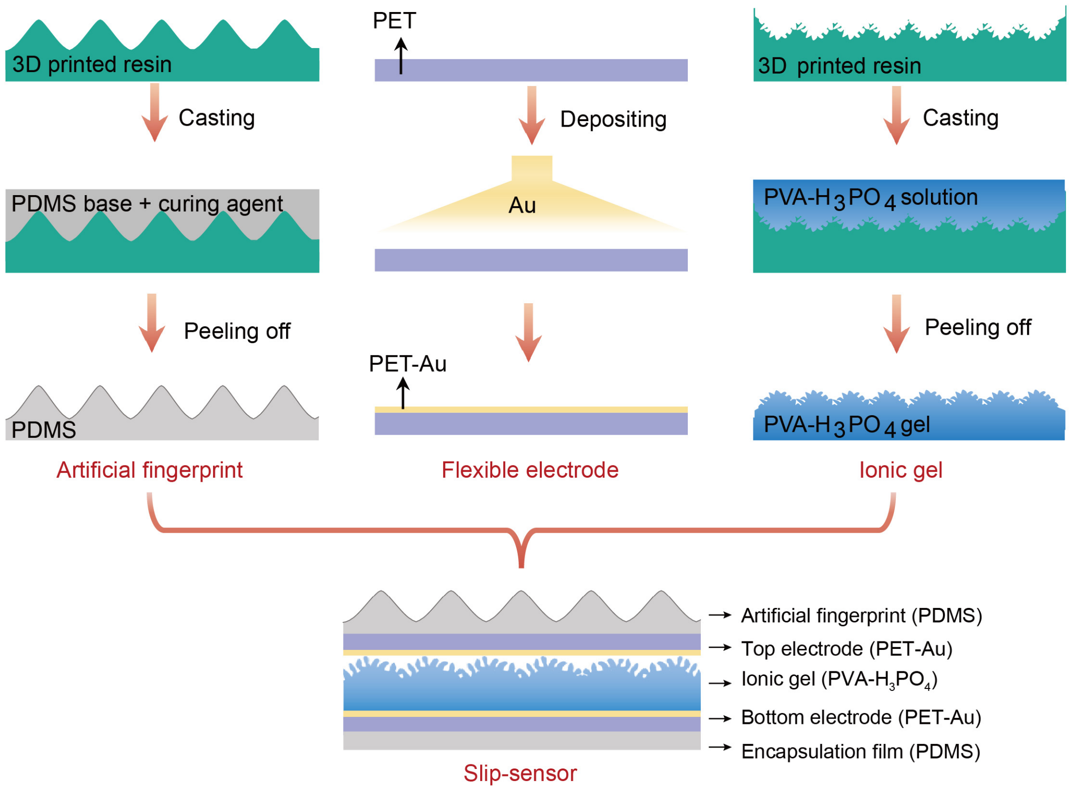

Supplementary Fig. 21. Schematic for the preparation of the slip-sensor. The fabrication involves in the preparation of the fingerprint, the electrode, and the ionic gel with graded microstructures.

The supplementary note: recognition of microstructure by human subjects

Five volunteer subjects were selected to recognize the fine microstructures with spacings of 10, 15, 20, 30, 40, and 50 μm together with a nonstructured surface. During the training and testing, the subjects were asked to wear eyeshades to avoid visual interference. Two copies of each sample were prepared, one set for training and other set for testing. Considering that subjects cannot remember the characteristics of identified samples for a long time, they were allowed to touch a sample from the training set searched for a match from the test set. Each identified sample were tested for 10 times by each subject, with a total of 350 tests in total by the 5 subjects. The recognition accuracy of each subject for the identified sample was determined by dividing the number of correct reports for each sample by the total number of reports for the sample. Finally, the average recognition accuracy of the five subjects for each sample was output to the confusion matrix as the final recognition result.
