# Supplementary material for: A robotic sensory system with high spatiotemporal resolution for texture recognition
Source: Nat Commun. 2023 Nov 14;14:7121. doi: 10.1038/s41467-023-42722-4 (PMC10645869; doi:10.1038/s41467-023-42722-4)
Supplement: Supplementary file 3 — Description of Additional Supplementary Files [file 41467_2023_42722_MOESM3_ESM.pdf]

Description of Additional Supplementary Files

Supplementary Movie 1: A portable and real-time sensory system for texture recognition using a prosthetic hand with a slip-sensor, operating at a consistent sliding rate of 2 $mm \cdot s^{-1}$.

Supplementary Movie 2: A portable and real-time sensory system for texture recognition using a slip-sensor integrated on a human hand, operating at random sliding rates.
